# A Genome-Wide Association Study in Large White and Landrace Pig Populations for Number Piglets Born Alive

**DOI:** 10.1371/journal.pone.0117468

**Published:** 2015-03-17

**Authors:** Sarah Bergfelder-Drüing, Christine Grosse-Brinkhaus, Bianca Lind, Malena Erbe, Karl Schellander, Henner Simianer, Ernst Tholen

**Affiliations:** 1 Institute of Animal Science, Department of Animal Genetics, University of Bonn, Bonn, Germany; 2 Biotechnology Research (FBF), Bonn, Germany; 3 Animal Breeding and Genetics, University of Goettingen, Goettingen, Germany; The University of Chicago, UNITED STATES

## Abstract

The number of piglets born alive (NBA) per litter is one of the most important traits in pig breeding due to its influence on production efficiency. It is difficult to improve NBA because the heritability of the trait is low and it is governed by a high number of loci with low to moderate effects. To clarify the biological and genetic background of NBA, genome-wide association studies (GWAS) were performed using 4,012 Large White and Landrace pigs from herdbook and commercial breeding companies in Germany (3), Austria (1) and Switzerland (1). The animals were genotyped with the Illumina PorcineSNP60 BeadChip. Because of population stratifications within and between breeds, clusters were formed using the genetic distances between the populations. Five clusters for each breed were formed and analysed by GWAS approaches. In total, 17 different significant markers affecting NBA were found in regions with known effects on female reproduction. No overlapping significant chromosome areas or QTL between Large White and Landrace breed were detected.

## Introduction

Reproduction traits of livestock are important because of the major role they play in the economic success of production [1]. The efficiency of pig production largely depends on the number of piglets born alive (NBA) and the number of piglets weaned (NPW). Up to the present, selection based on traditional breeding programmes using Best Linear Unbiased Prediction (BLUP) has been successful in improving maternal reproductive traits such as NBA. However, genetic improvement of female reproduction traits is difficult and complex because of low heritability and sex limited expression and because phenotyping is only possible late in a sow’s life. These conditions constitute a challenge for traditional animal breeding programmes. The exploration of the genetic architecture of reproduction traits is necessary because of the complex genetic and biological processes involved [1,2].

Since the very beginning of quantitative trait loci (QTL) mapping [3], about 10,000 QTL for 653 different traits have been identified in the pig genome (PigQTLdb, http://www.animalgenome.org/cgi-bin/QTLdb/SS/index, [4]). Most of the reported QTL affect production and meat quality traits. For reproduction traits, 137 QTL were identified for total number born, 110 QTL for body weight at birth and 106 QTL for NBA (July 2014).

Several studies have investigated the biological foundation in regard to the high impact of NBA on pig production. Genes such as retinol binding protein 4 (*RBP4*), estrogen receptor 1 and 2 (*ESR1*, *ESR2*) and porcine insulin-like growth factor 2 (*IGF2*) were identified to be positively associated with NBA [1,5–7], but these genes explain only a relatively small proportion of the genetic variation of NBA.

In the past, genome-wide scans using microsatellites were performed to identify regions affecting the potentially interesting traits. The development of the PorcineSNP60 BeadChip [8] allows the detection of QTL and candidate genes in a higher resolution. In a recent study Onteru et al. [9] have detected novel QTL regions for pig reproduction traits which do not overlap with QTL intervals previously reported using microsatellites.

In Europe, the two breeds Large White (LW) and Landrace (LR) are typical dam lines in commercial pig breeding programmes. However, differences between the two breeds were found in several studies which investigated reproduction traits such as NBA. For example, it was shown that LW sows had slightly higher NBA compared to LR sows [10–12]. Moreover, most breeding companies have their own LW and LR populations with different breeding objectives. Breeding stock is not normally exchanged between organisations. This leads one to expect differences between the breeding companies and their breeding stock.

In order to map QTL affecting NBA, genome-wide association studies (GWAS) were performed in LW and LR populations of different breeding companies located in Germany, Switzerland, and Austria. The aims of the study were

- to reveal genetic similarities and differences between LW and LR populations of different breeding organisations,
- to identify significant associated SNPs for NBA, and
- to clarify the biological relevance of these significant markers.

## Materials and Methods

### Animals and phenotype data

**Fig 1:**
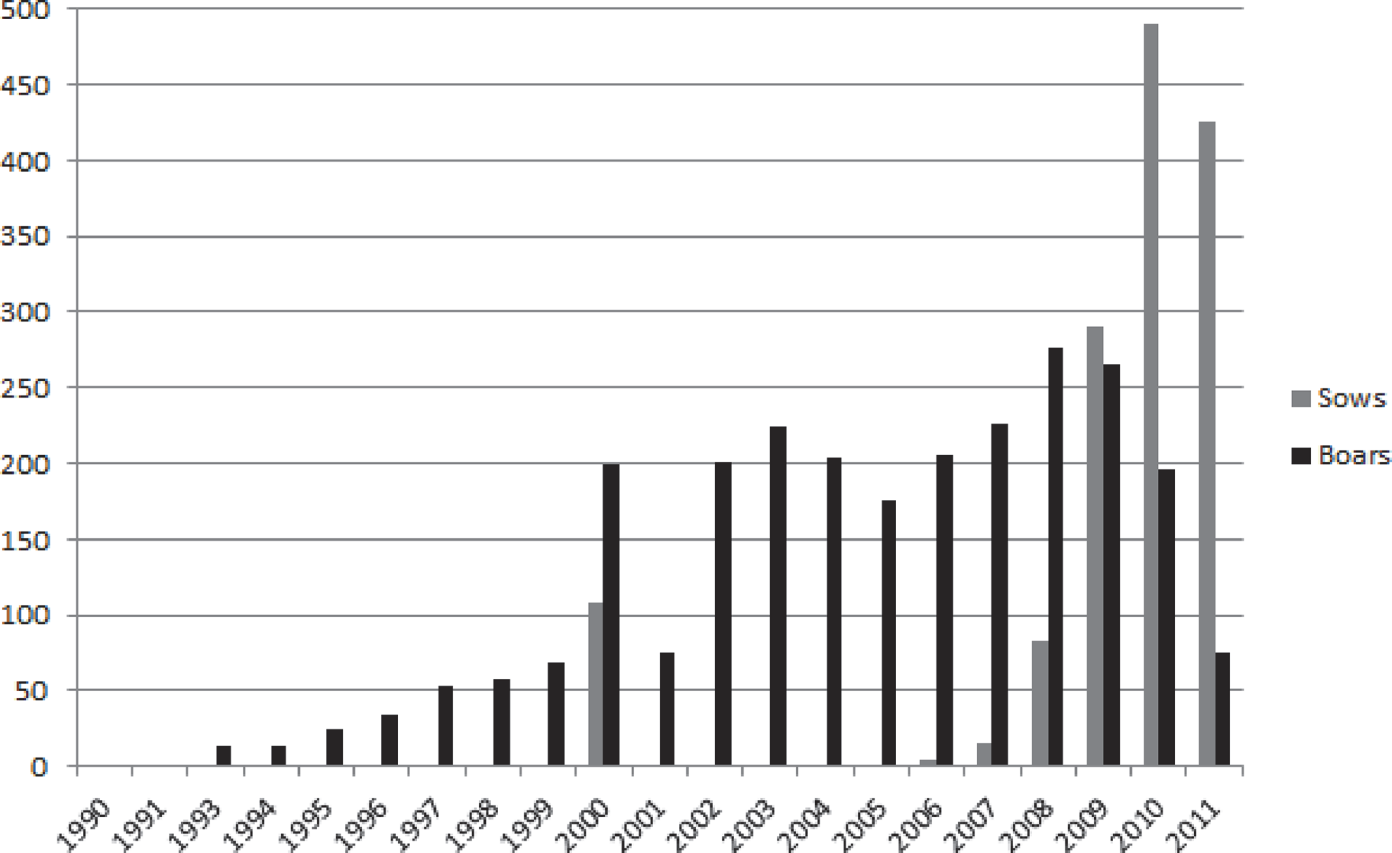
Frequencies of years of birth from all animals by gender.

The study included a total of 4,012 LW and LR pigs from herdbook and commercial breeding companies across Germany (3), Austria (1) and Switzerland (1). Data of 2,365 (boars: 1,435, sows: 930) LW and 1,647 (boars: 1,159, sows: 488) LR animals born between 1990 and 2011 were recorded (Table 1). The frequencies of years of birth of all animals are shown by gender in Fig. 1. Breeding values for NBA were routinely estimated by the breeding companies using a standard animal repeatability model and were provided for the study.

**Table 1:** Number of genotyped animals.

	**Landrace**			**Large White**		
**Country**	**N**	**Boar**	**Sow**	**N**	**Boar**	**Sow**
Germany	**1288**	925	363	**1146**	790	356
Austria	**266**	141	125	**191**	148	43
Switzerland	**93**	93	-	**1028**	497	531
**Σ**	**1647**	1159	488	**2365**	1435	930

### SNP Quality control

Tissue samples (semen or hair follicle) of the pigs were genotyped with the Illumina PorcineSNP60 Bead Chip [8] in the laboratory Life & Brain GmbH, Bonn.

SNPs were excluded from further analysis under the following conditions: a) Minor allele frequency (MAF) < 0.5%, b) Call rate < 95% and c) strong deviation from the Hardy-Weinberg-Equilibrium (p < 10^−3^). Quality control was performed as implemented in the GenABEL package [13] within defined population clusters.

### Population structure

GWAS were performed within breeds (LW or LR) and clusters comprising different sub-populations. In order to visualize possible population stratifications, multidimensional scaling (MDS) plots of an identity-by-state (IBS) matrix were generated containing the two most important principal components of the underlying genetic variation. These two-dimensional MDS plots of the IBS matrix revealed the overall genetic distances between the animals. Based on the visualized genetic distances, animals of the LW and LR populations were analysed separately. In addition, four sub-populations were identified within the LW and LR breeds. Additional GWAS were performed within these clusters, which comprise animals from one to four different breeding organisations.

### Genome-wide association study

The GWAS were based on an combined approach developed by Amin et al. [14] and Price et al. [15] and implemented in the R-Package GenABEL [13,14,16]. In order to control population stratification the “Genome-wide Rapid Analysis using Mixed Models and Regression” (GRAMMAR) [14] combined with EIGENSTRAT [15] was used. A similar, combined procedure was suggested by Zhao et al. [17].

In a first step, the phenotypic data (breeding values) were corrected for the fixed effect “breeding organization” and a polygenetic effect *(a)* by means of Equation (1):

$$y^{*}=y-(\mu +X\beta +Za)$$ (1)

with *y* ^*^ and *y* as vectors of pre-corrected and original estimated breeding values (EBVs), respectively, *β* as solution vector of the fixed effect ‘breeding organisation’, and *a* as random additive polygenic *(a*_*i*_ *∼ N (0*,*G×σ* ^*2*^_*a*_ *))* effect, which estimates the contribution from the polygene (breeding value) with *G* as the genomic kinship matrix and the additive genetic variance *σ* ^*2*^_*a*_. *X* and *Z* are the corresponding design matrices for the fixed and random effects.

The genomic kinship (G_ij_) was estimated by applying the method suggested by Astle & Balding [18]:

$$G_{ij}=\frac{1}{L}\sum_{l=1}^{L}\frac{\left(g_{l,i}-p_{l})(g_{l,j}-p_{l}\right)}{p_{l}(1-p_{l})}$$ (1)

with *L* as the number of SNP, *p*_*l*_ as the allelic frequency at *l*-th locus (major allele) and *g*_*l*,*j*_ / *g*_*l*,*i*_ as the genotype of *j*-th / *i*-th individual at the *l*-th locus, coded as 0, 1/2 and 1, corresponding to the rare homozygous, heterozygous, and common homozygous genotype.

Ignoring the covariance between animals from one family can lead to a high number of false-positive SNPs. The residuals computed with GRAMMAR are corrected for polygenic relationships between the animals and can be used as a new phenotype in association analyses [14,16].

In a second step, these familial correlation-free residuals were included in a simple linear regression as new phenotype for association test (2):

$$y^{*}=\mu +kg+e$$ (2)

with *y* ^*^ as the vector pre-corrected EBVs from (1), *μ* as the mean, *g* is the vector of genotypes at the marker, *k* as the marker genotype effect and *e* as the vector of random residuals.

In order to verify remaining population stratification, the inflation factor *λ*, which depends on the squared original test statistic of the *i-th* SNP ($T_{i}^{2}$) was calculated as

$$\lambda =\frac{Median(T_{i}^{2})}{0.4549}$$

Aluchenko et al.[13] and Price et al. [19] showed that an inflation factor *λ* in the range of 1.0 to 1.05 is an indicator of a sufficiently corrected population stratification which can be analysed with an acceptable risk of false positive results. Preliminary results of our analysis showed that *λ* deviates considerably from this optimum. This implies that serious population stratifications still exist.

In order to correct for this problem, model 2 was extended by principal components (PC) estimated from the genomic kinship (EIGENSTRAT) [13,15] which were included as fixed covariables. The genomic kinship matrix was used to reveal the PC reflecting the axes of genetic variation and describing the stratification of the populations involved in this study. These PC were used to adjust the phenotype and the genotype for population stratification. The estimation of the PC and the association analysis was performed with the function ‘egscore’ as implemented in the R-package GenABEL [13].

The number of PC used in this step is variable and depends on the ability to correct different levels of population stratifications. The number of PC was increased stepwise and after each step the level of population stratification was quantified via the inflation factor *λ*.

The final number of PC was chosen so that the inflation factor *λ* [20] was nearest to 1.

The inflation factor *λ* and the observed versus the expected p-values for each SNP are illustrated in quantile-quantile (Q-Q) plots for each cluster. Two regression lines are fitted which represent the optimal (*λ* = 1) and the calculated inflation factor *λ*. In case of unstratified population structures, no visible differences can be observed between the two regression lines.

In order to reduce the risk of false-positive associations, the p-values of the SNP significance tests were corrected using the Bonferroni-adjustment. Thresholds for genome-wide and chromosome-wide significance levels were 5%.

Variance of the pre-corrected EBVs (σ^2^_y*_) explained by each SNP was calculated approximately using following formula:

$$r^{2}=\frac{\chi_{1df}^{2}}{N-2+\chi_{1df}^{2}}$$ (3)

with *χ* ^*2*^_*1df*_ as the test statistic for each SNP resulted from association test and *N* as the number of animals. This formula resulted from the transformation of a student’s t-distribution into a z-distribution [21]. In our analysis, *r* ^*2*^ cannot be interpreted as the proportion of explained phenotypic variance of NBA—as is usually the case—, because pre-corrected EBVs were analyzed instead of phenotypes. However, *r* ^*2*^ might be a rough indicator of the explained proportion of the additive genetic variance of NBA and could be used to rank the importance of QTL only.

Pig Sscrofa 10.2 (International Swine Genome Sequencing Consortium) [22] was used to annotate the significant associated SNPs. The search for biologically relevant genes was performed with Ensembl BioMart [23,24]. For that, a 2 Mb window around a significant region was chosen.

## Results

### Population structure analysis

**Fig 2:**
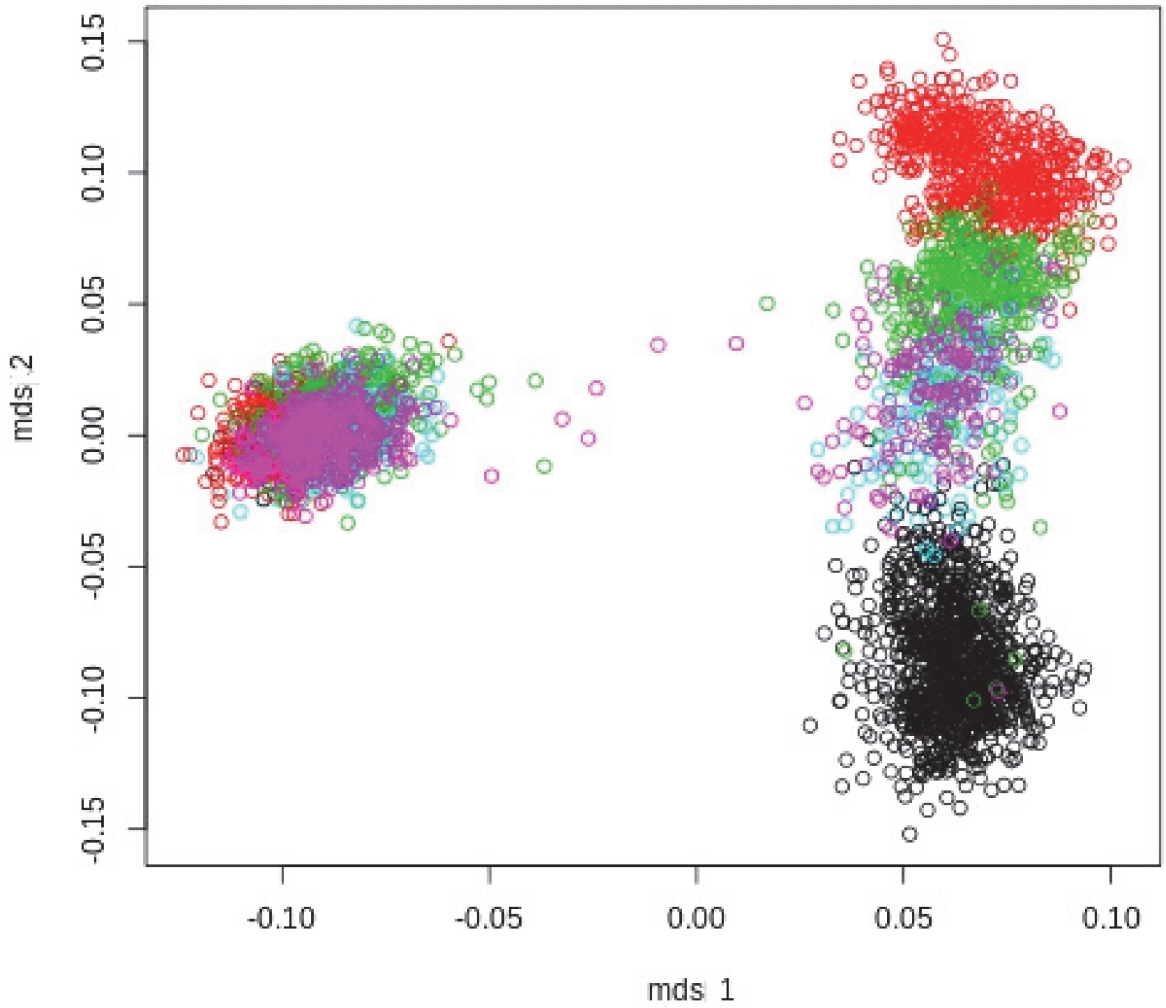
MDS Plot of Landrace (left) and Large White (right) populations of 5 European breeding companies.

**Fig 3:**
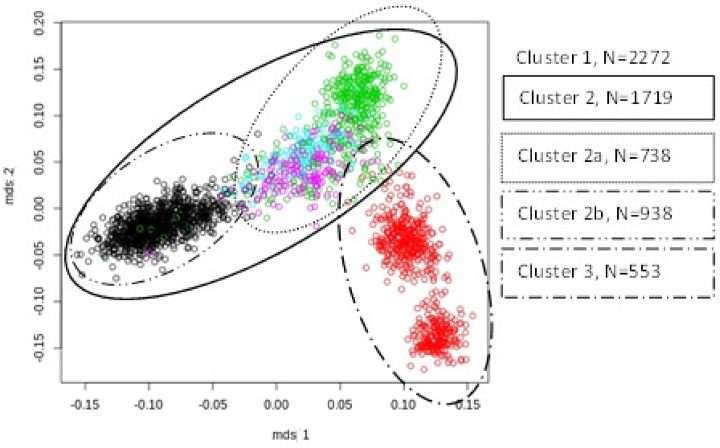
MDS plot of Large White population, each colour represents one breeding company, circles show two different clusters.

**Fig 4:**
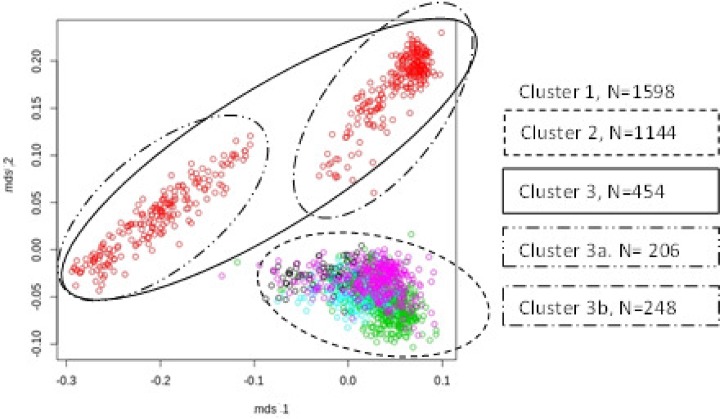
MDS Plot of Landrace population of 5 European breeding companies, circles indicate different clusters.

MDS plots were used to visualize the genomic distances between the animals (Figs. 2, 3, and 4). Fig. 2 revealed that the breeds LW and LR had a large genetic distance and should be regarded as more or less genetically disconnected. Each breed was analysed separately because of distinct genetic differences between LW and LR. Additionally performed visual inspections of the breed specific MDS plots of LW and LR populations led to various cluster definitions (Figs. 3, 4).

The animals of the breed LW (LW_1) were grouped into four sub-clusters (Fig. 3). Cluster LW_3 and LW_2b contained only animals of one breeding organisation, whereas cluster LW_2a covered genetically overlapping pigs of three breeding organisations. In addition, cluster LW_2 combined the clusters LW_2a and LW_2b, which overlap only to a small extent.

In the LR population (cluster LR_1) four sub-clusters were assigned (Fig. 4). Cluster LR_2 was formed by excluding the breeding company (cluster LR_3) with the highest deviation from the LR_1 dataset. In addition, two distinct sub-populations were extracted from cluster LR_3 which form cluster LR_3a and LR_3b.

### Quality control

SNP quality control was performed within the various clusters. The quantity of remaining genetic markers lay between 39,408 and 45,303 (LW) and 42,205 and 46,066 (LR) clusters. The number of animals ranged between 553 and 2,272 for LW or 206 and 1,598 for LR clusters. More detailed information about each cluster is given in Table 2.

**Table 2:** Dataset and results of association analyses.

	**Dataset**		**Association analyses** *				
**Data set**	**N animal**	**N marker**	**PC**	**λ**	**Chromosome (Genome)-wide significant SNPs**	σ^2^_y*_ **(%)**	**MAF**
LW_1	2272	39408	372	1.004	3 (0)	0.7–0.9	0.8–21.1
LW_2	1719	43216	256	1.005	5 (0)	1.1–1.4	0.5–22.5
LW_2a	738	45242	74	1.002	4 (3)	2.4–4.6	0.6–21.2
LW_2b	938	45303	151	1.004	2 (0)	1.8–2.1	16.3–17.6
LW_3	553	43549	109	1.004	0 (0)	-	-
LR_1	1598	42721	293	1.004	2 (0)	1.1–1.3	31.4–39.4
LR_2	1144	46066	185	1.001	0 (0)	0	0
LR_3	454	42205	76	1.009	2 (0)	4.2–4.8	1.2–3.7
LR_3a	206	43416	26	1.015	0 (0)	-	-
LR_3b	248	44013	22	1.009	1 (0)	8.0	2.2

* = Numbers of chromosome-wide and genome-wide significant associated SNPs with NBA (p>0.05%); PC = number of principal components; λ = inflation factor; MAF = minor allele frequency; σ^2^_y*_ = Variance of the pre-corrected EBVs.

### Influence of population stratification

**Fig 5:**
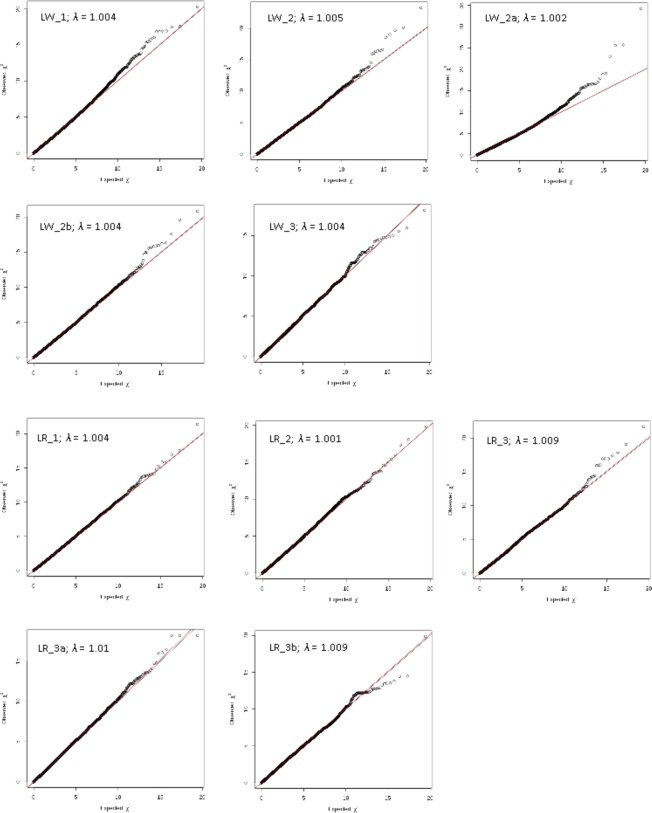
Q-Q plots of all association studies for all breed clusters.

In order to ensure the power and accuracy of GWAS, it is essential to take possible population stratifications [13,25,26] into consideration. Therefore, associations between SNP and NBA were estimated within the genetically more or less overlapping clusters. In addition, PC which condensed the genetic relationships between the animals was used in the statistical model as covariates to correct for existing population stratification. Depending on the cluster, different numbers of PC were required in order to avoid negative effects of population stratification on the validity of the GWAS analysis. The number of PC used in the analyses of various clusters ranged from 22 (LR_3b) to 372 (LW_1). Genomic inflation factors in all clusters were close to one (Table 2). Cluster specific Q-Q plots (Fig. 5) contain regression lines which were calculated by a linear regression of expected test statistics (independent variable) on observed test statistic (dependent variable). The slopes of these lines correspond to the calculated inflation factor, which is close to 1 in all clusters analysed. This shows that possibly existing stratifications of the populations do not adversely affect the validity of corresponding GWAS analysis.

### Genome-wide association analyses

**Fig 6:**
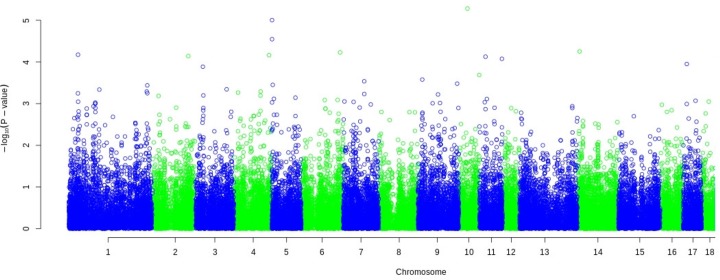
Manhattan plot of genome wide association study for NBA in LW_1.

The Manhattan plots show the p-values of the SNP association test for the target trait NBA ordered according to the genomic positions (representative by Fig. 6; S1 Fig., S2 Fig., S3 Fig., S4 Fig., S5 Fig., S6 Fig., S7 Fig., S8 Fig., and S9 Fig.). 14 different chromosome-wide and three genome-wide significant SNPs were detected in the analysed clusters. Three of these SNPs had a MAF below 1%.

SNPs which were significant in both breeds or in different clusters containing animals from different breeding organisations would have been of particular interest. However, no significant markers or chromosome regions were found to be shared by the breeds. Moreover, only a small number of SNPs were found to be identical in the different clusters of each breed. These SNPs and cluster specific significant markers will be described in the following sections.

**Large White.** In LW_1 three chromosome-wide significant markers were found on SSC5 and SSC10. Each of these markers explained less than 1.0% of σ^2^_y*_. The population LW_1 was subdivided into clusters LW_2 (animals from four breeding organisations) and LW_3 (one breeding organisation). In LW_2 and LW_3 no genome-wide significant SNPs were found. However, within cluster LW_2 five QTL were detected on a chromosome-wide significant level. Each of these QTL explained between 1.1 to 1.3% of σ^2^_y*_ of the target trait NBA (S1 Fig. and S4 Fig., Tables 2 and 3). Because of a smaller degree of genetic overlapping, LW_2 was further subdivided into clusters LW_2a and LW_2b. The analyses of these clusters revealed three (LW_2a) and two (LW_2b) chromosome-wide significant SNPs for NBA (Tables 2 and 3), which explained 2.4 to 4.6% and 1.8 to 2.2% of σ^2^_y*._ Three of the QTL detected in dataset LW_2a were significant on a genome-wide level (S2 Fig., S3 Fig.). One of the significant SNP associations on SSC9, identified in cluster LW_2, was confirmed by the analysis of sub-cluster LW_2a. Additionally, three SNPs which were found on SSC5 and SSC10 in LW_1 were also identified in LW_2. This was to be expected, because LW_2 is a subset of the larger cluster LW_1 and LW_2a is one of LW_2.

**Table 3:** Statistic of significant SNPs in LW.

**SNP**	**SSC**	**N** ^A^	**MAF** ^B^	**Var** ^C^	**p-value** ^D^	**Cluster**
ALGA0018160	3	738	2.0	3.4	0.001**	LW_2a
ASGA0023685	5	2272	20.9	0.8	0.02	LW_1
	5	1719	22.3	1.2	0.01	LW_2
MARC0103593	5	2272	21.1	0.8	0.05	LW_1
	5	1719	22.5	1.1	0.03	LW _2
MARC0104982	5	738	0.6	2.5	0.03	LW _2a
ALGA0055303	9	1719	0.6	1.3	0.004	LW _2
	9	738	0.6	4.6	<0.001**	LW _2a
ASGA0046811	10	738	21.3	2.6	0.02	LW _2a
MARC0070030	10	2272	0.8	0.9	0.007	LW _1
	10	1719	0.8	1.1	0.01	LW _2
MARC0043480	10	938	16.3	2.2	0.01	LW _2b
DRGA0010601	10	938	17.6	1.9	0.04	LW _2b
ASGA0090608	10	738	5.7	2.4	0.05	LW _2a
H3GA0030853	11	1718	1.9	1.1	0.03	LW _2
MARC0006510	11	738	0.6	3.0	<0.001**	LW _2a
ASGA0079878	18	738	0.6	3.5	0.0004**	LW _2a

^A^ Number of analysed animals,

^B^ minor allele frequency (MAF),

^C^ σ^2^_y*_ = Variance of the pre-corrected EBVs (Var, %),

^D^ nominal p-value and corresponding significant thresholds:

**genome-wide significant (p_gem_ < 0.05).

**Landrace.** In the data set LR_1 two SNPs reached the chromosome-wide significance threshold of 5% (SI 5, Tables 2 and 4). These associations were located on chromosome 9 and 11, they explained up to 1.3% of σ^2^_y*_. After visual inspection of the MDS plots, LR_1 was subdivided into clusters LR_2 and LR_3 which contained 4 or 1 breeding organisations, respectively. In the case of LR_2, no SNP reached the genome- or chromosome-wide significance level (S6 Fig., Tables 2 and 4). On the other hand, association test performed for cluster LR_3 resulted in two SNPs with chromosome-wide significance, explaining up to 4.8% of σ^2^_y*_. These significant SNPs were located on SSC 7 and SSC16 (S7 Fig., Tables 2 and 4). Although cluster LR_3 contained only animals from one breeding organisation, two genetically disconnected sub-clusters (LR_3a and LR_3b) were identified. Association tests in LR_3a resulted in no significant SNPs. For LR_3b and LR_3, one marker located on SSC16 reached the chromosome-wide significance level and explained up to 8.0% of σ^2^_y*_ (S9 Fig., Tables 2 and 4).

**Table 4:** Statistic of significant SNPs in LR.

**SNP**	**SSC**	**N** ^A^	**MAF** ^B^	**Var** ^C^	**p-value** ^D^	**Cluster**
CASI0006750	7	454	3.7	4.2	0.04	LR_3
MARC0070952	9	1598	31.3	1.3	0.01	LR _1
H3GA0030985	11	1598	39.4	1.1	0.05	LR _1
ASGA0072103	16	454	1.2	4.8	0.005	LR _3
	16	248	2.2	8.0	0.01	LR _3b

^A^ Number of analysed animals,

^B^ minor allele frequency (MAF),

^C^ σ^2^_y*_ = Variance of the pre-corrected EBVs (Var, %),

^D^ nominal p-value and corresponding significant thresholds.

## Discussion

### Population stratification

In the present study, a combined GWAS-approach was used to identify QTL influencing NBA in two maternal pig breeds. When analysing such large scale heterogeneous data, it is of major importance to correct for potential population stratifications in order to ensure the accuracy of the statistical analysis. Several studies have shown that ignoring population stratification will lead to an inflation of false positive QTL and to a loss of statistical power [13,25,26]. In order to avoid such negative effects, our study analysed several clusters compromising animals from only one or from genetically overlapping breeding organisations. As a first result, it was found that animals of the LW and LR breed in the present study do not genetically overlap. This can be seen in the corresponding MDS plot (Fig. 2). For this reason both breeds were analysed separately. In addition, sub-clusters within the two breeds were identified. These sub-clusters are presumably the result of the different selection strategies used by the different breeding organisations. Sub-populations from a limited number of breeding organisations were investigated to identify common regions affecting the target trait NBA. This is a generally accepted procedure and has been utilised in several GWAS in pigs and cattle [27,28].

The defined clusters were statistically evaluated with an approach that combines the GRAMMAR [14] und EIGENSTRAT [15] methods. Within the GRAMMAR approach estimated breeding values for the trait NBA are pre-corrected for the effects ‘breeding organisation’ and ‘familial correlations’, taking into account the genomic “true” relationship between animals. This approach has two advantages: a) the genomic kinship matrix shows the true proportion of shared alleles whereas a pedigree based kinship matrix displays the expected proportion and b) familial correlations are removed from the new phenotype by calculating environmental residuals for association test [14,29]. This is especially important for analysing EBVs as dependent variables because in this case distinct correlations between the EBVs of relatives can be expected. Despite these corrections, the inflation factor, which was calculated according to model 1 (GRAMMAR approach), deviates considerably from the optimum of λ = 1 in each cluster. Therefore, in the second part of the combined approach (EIGENSTRAT), the detection of QTL is based on a model which includes a number of genomic PC depending on the cluster as fixed covariates. This method (EIGENSTRAT) has been applied in several other studies [26–28,30]. The PC condenses the genomic covariance structure of the animals into a series of factors with decreasing importance. The PC act as a correction factor for possible population stratification, but on the other hand, they also reduce the genetic variation which can be used to detect QTL. Although this method leads to an efficient elimination of population stratification, it remains unclear if the inclusion of a high number of PC (>10) leads to an unacceptable loss of utilizable genetic variation. This might have a considerable impact on the power of the association tests [25,28]. In order to balance the two conflicting objectives—removal of population stratification and retention of utilizable genetic variation—, we increased the number of PC stepwise until an acceptable solution was found. The effects of increasing the number of PC were monitored by evaluating the level of the inflation factor *λ*, which is an indicator of the remaining population stratification. Generally, a value of *λ* between 1.00 and 1.05 is regarded as tolerable [13,19]. This acceptable range was reached in all analyses after the inclusion of 22 to 372 PC. Aulchenko et al. [13] suggested including 10 PC in the GWAS model in human, which can be regarded as a compromise between correcting for population stratification and retention of utilizable genetic variation. As expected, the number of significant markers increased substantially when 10 PC were used. However, the inflation factors in all analysis were below one, so that the results were not further interpreted.

### Minor allele frequency

In GWAS, SNPs with a MAF lower than 1% are frequently excluded from the data set. In the present study a threshold of 0.5% was chosen instead, which can be justified by the findings of Tabangin et al. [31] and Stephens & Balding [32]. Tabangin et al. [31] found that rare SNPs did not show significantly higher false-positive results than common SNPs. They concluded that the removal of SNPs with a low MAF would not be necessary to reduce false-positive results. Stephens & Balding [32] pointed out that the consideration of the p-value alone is not sufficient to characterize the association between the SNP and trait. The statistical power in association tests is of high importance in order to quantify the true dimension of the association. This power is influenced by the MAF and is reduced when SNPs with low MAFs are removed [32,33].

Only five out of a total 17 significant SNPs in the present study had a MAF of < 1%. These SNPs were located in regions where trait specific QTL or genes have been mapped (Tables 3, 4, and 5). Their physiological role could indicate a functional relevance regarding the variation of the trait examined here. Gorlov et al. [33] and Cargill et al. [34] found in their analyses that the proportion of functional SNPs was highest among SNPs with a low MAF. The elimination of rare SNPs could thus decrease the potential for genetic improvement when using genomic selection in animal breeding.

**Table 5:** Results of annotation for all analyses with previously reported candidate genes, QTL or association in SNP region.

**SSC**	**SNP**	**Position (Mbp)**	**Genes in SNP Region** *	**Previously reported QTL or Associations in SNP region** **	**Cluster**
3	ALGA0018160	27925965	-	NBA, CLN	LW_2a
5	ASGA0023685	876762	*PPARα*, *Fbln1*	NSB	LW_1,LW_2
5	MARC0103593	961240	*PPARα*, *Fbln1*	NSB	LW_1,LW_2
5	MARC0104982	91550413	*VEZT*	-	LW_2a
7	CASI0006750	115511369	*FLRT2*	-	LR_3
9	MARC0070952	14861213	-	TNB	LR_2
9	ALGA0055303	139041276	*PTGS2*,*PLA2G4A*	CLN	LW_2,LW_2a
10	ASGA0046811	18203672	*AHCTF1*	-	LW_2a
10	MARC0070030	32526661	-	CLN	LW_1,LW_2
10	MARC0043480	63867699	*ITGB1*	CLN, FSH	LW_2b
10	DRGA0010601	63869377	*ITGB1*	CLN, FSH	LW_2b
10	ASGA0090608	76815569	*CTSL*	-	LW_2a
11	H3GA0030853	82720	-	-	LW_2
11	H3GA0030985	3733271	*FLT1*	-	LR_1
11	MARC0006510	74240078	-	NSB	LW_2a
16	ASGA0072103	6470509	-	NBA	LR_3,LR_3b
18	ASGA0079878	47312409	-	NBA	LW_2a

SSC = Sus scrofa; TNB = total number born; NBA = number born alive, NSB = number of stillborn piglets; CLN = corpus luteum number, FSH = plasma follicle-stimulating hormone.

* The declaration of gene symbols can be obtained from Ensembl or http://www.ncbi.nlm.nih.gov/gene.

** The QTL information was obtained using http://www.animalgenome.org/cgi-bin/gbrowse/pig/.

### Significant markers for NBA: Across population

In LW, SNPs significant across sub-populations were found in the analysis of clusters LW_1 and LW_2 as well as in LW_2 and LW_2a, which had a certain proportion of animals in common contain shared proportions of identical animals. A remarkably low number of QTL were found in the genomically homogeneous cluster LW_2b, which consists of animals from only one breeding organisation. The high number of PC (151) with negative impact on the utilizable genetic variation might explain this result. In addition, the year of birth of the pigs from this breeding organisation covers the years 1990 to 2011 (Fig. 1). This long period of selection might influence the frequency of important genes and/or the linkage phase between marker and QTL, but not necessarily the genomic population structure displayed by the MDS plots (Figs. 2 and 3).

The LR population of one breeding organisation (LR_3) was genetically disconnected, so that two sub-clusters (LR_3a and LR_3b) were formed and analysed separately. The genetic disconnection can be explained by the import of breeding animals into this breeding organization in the past. Within the different LR clusters, only one SNP located on SSC16 was found in two clusters, LR_3 and its subset LR_3b.

### Significant markers for NBA: Position and biological relevance

Detailed information about significant SNPs and the results of annotation for all analyses with previously reported candidate genes, QTL or association in SNP regions are given in Table 5.

In the analysis of LW_2a, one SNP significantly associated with NBA on SSC3 at 27.9 Mb was located within a region where QTL have been found for NBA and ovulation rate (OR) in previous studies [9,35] (Table 5). Up to the present, no gene with an influence on these reproductive traits has been located in this chromosome region.

At the distal end of the p-arm of SSC5 two significant markers (ASGA0023685, MARC0103593) were found in LW_1 as well as in LW_2 (Table 5). In the cluster LW_2b, these two markers slightly exceeded the 5% significance threshold. The gene peroxisome proliferator activated receptor α (*PPARα*), which is part of a nuclear hormone receptor family, was mapped within the 2 Mb window around these marker positions. In Polish LR and Pietrain, it has been shown that the expression of *PPARα* is significantly higher in endometrial tissue at early stage of pregnancy than during the estrous cycle [36]. Gene expression was lower at day 10–12 and 22–30 of pregnancy when the maternal recognition of pregnancy and the end of the implantation of the fetus in the endometrium take place. The study concluded that *PPARα* is involved in these two important events. A second gene (Fibulin-1, *Fbln1*), involved in building blood vessel walls, is located at 1.07–1.16 Mb on SSC5 (Table 5). The importance of this gene was illustrated by a perinatal mortality of mice with homozygous knock-out phenotype [37]. Vezatin (*VEZT*) was located at 92.2 to 92.3 Mb which was next to the found marker at 91.5 Mb on SSC5 when analyzing cluster LW_2a. The physiological role of *VEZT* has not been established in pigs, but Hyenne et al. [38] reported a function of *VEZT* during preimplantation of mice embryos. They inhibited the expression of this gene and found developmentally arrested embryos with limited cell-cell interactions which failed to form a young blastocyst. This finding underlines the potential importance of *VEZT* for maternal reproduction.

In cluster LR_3, one chromosome-wide significant marker (CASI0006750) was found at 115.5 Mb on SSC7 with a MAF of 2%. Fibronectin leucine-rich repeat transmembrane protein (*Flrt2*) was mapped close to this marker (114.35–114.36 Mb) (Table 5) which is involved in the embryonic development of the heart. Mice homozygous null embryos were developmentally arrested and died at mid-gestation caused by cardiac insufficiency [39].

At position 14.8 Mb the marker MARC0070952 was found on SSC9 in LR_1 and in LR_2, but in LR_2 the marker exceeds the chromosome-wide 5% p-value threshold only by a small amount (p = 5,5%). In pigs, Onteru et al. [9] detected one QTL affecting TNB in this region (Table 5). Up to the present, no genes with an influence on reproduction in pigs have been identified in this chromosome region. A second detected marker on SSC9 was found in the overlapping clusters LW_2 and LW_2a (ALGA0055303, 139.0 Mb) with a genome-wide significance in LW_2a although the MAF was below 1%. In a previous study, QTL for corpus luteum number have been detected in this chromosome region of SSC9 [35]. Additionally, prostaglandin-endoperixode synthase 2 (*PTGS2*, also known as cyclooxygenase II), was mapped in this area of SSC9 (140.2–140.3 Mb) (Table 5). *PTSG2*-null mice showed defects in the mentioned reproduction traits [40,41], e.g. implantation failure [41]. Ashworth et al. [42] investigated the role of *PTSG2* in the estrous cycle and early pregnancy of pigs. They concluded that this gene has an impact on placental attachment and embryo survival in pigs. An early estrogen exposure at the beginning of the pig’s pregnancy leads to an altered *PTSG2* expression. This could be one of the reasons for a total embryonic loss during implantation due to endocrine disruption of pregnancy [42]. Additionally, it has been shown that *PTGS2* is important for the regulation of ovulation and fertilization which determine the number of preimplanted embryos [41,43,44] and therefore influences litter size in pigs. Phospholipase A_2_ group 4A (*PLA*_*2*_ *G4A*) is required for a normal *PTGS2* induction [45,46]. *PLA*_*2*_ *G4A* is also mapped in the chromosome region of the significant associated marker, which was found in LW_2 and LW_2a on SSC9 (140.4–140.6 Mb) (Table 5). Knocking out this gene leads to reduced litter sizes in mice caused by defects during implantation [47–51]. Kurusu et al. [52] also found a significantly reduced number of oocytes and preimplanted embryos in *PLA*_*2*_ *G4A* ^-^/^-^ mice in comparison to *PLA*_*2*_ *G4A* ^+^/^+^ mice leading to a reduction in litter size.

The SNP ASGA0046811 at position 18.2 Mb on SSC10 was significantly associated with NBA in LW_2a. The gene AT hook containing transcription factor 1 (*AHCTF1* also known as *ELYS*), was mapped close to this marker (17.3–17.4 Mb) (Table 5). The function of this gene in pigs is not clarified yet. Okita et al. [53] showed that *AHCTF1* deficient mice with a homozygous genotype for this mutation died after implantation phase. They observed impaired proliferation of the inner cells of the embryos and concluded that this gene is an important factor for the proliferation and survival of the inner cells and thus for the survival of the mouse embryo [53]. SNP MARC0070030 mapped on SSC10 at 32.5MB was found in LW_1 and LW_2, but had a MAF below 1% in both sub-populations. This marker is located in a previously described QTL for corpus luteum number [35] which is one of the main factors influencing NBA [54]. In the upstream chromosome region of SSC10, the SNPs DRGA0010601 and MARC0043480 (63.8 Mb) were associated with NBA in LW_2b. QTL affecting ovulation rate and plasma follicle-stimulating hormone (*FSH*) concentration were detected within that chromosome region in previous studies [35,55] (Table 5). In addition, integrin β 1 (*ITGβ1*) was mapped close to these markers (61.4–61.5 Mb). It has been shown that the G allele of *ITGβ1* has an effect on litter size in LW and LR [56]. Cathepsin L1 (*CTSL1*, at 76.9–77.0 Mb) is located close to the significant marker which was identified at position 76.8 Mb and was found to be associated with NBA in LW_2a. In pigs, this gene has the function of regulating the transport of macromolecules between mother and embryo. This is essential for the nutrition and development and thus the survival of the embryo [57].

On the p-arm of SSC11 one marker was found to be associated with NBA in LW_2. This is the first time that a QTL for NBA has been reported in this region. The chromosome-wide significant SNP H3GA0030985 was found at position 3.7 Mb in LR_1. The FMS-like tyrosine kinase 1 (*Flt1*) gene, which is one of the two receptors for vascular endothelial growth factor (*VEGF*)-A [58], was mapped at 5.3–5.5 Mb. It has a major impact on embryonic vascular development and on the cyclic blood vessel proliferation in the female reproduction tract [59]. An adequate vascular development is a key factor for the fetal-maternal exchange of nutrients, gases and wastes [60]. It has been shown that a targeted change of *VEGF-A* in mice leads to embryonic death [61,62]. Fong et al. [63] found that the gene *Flt1* has an essential function in embryonic vasculature. This was underlined by the fact that mutant mice homozygous in the *Flt1* locus did not survive the embryonic stage. Death was caused by abnormal vascular channels which these mutant embryos had developed. Furthermore, Ferrara et al. [59] suggested that *Flt1* appears as a “decoy” receptor for *VEGF-A* agonist during embryogenesis. In LW_2a, one marker was found on the q-arm of SSC11 in the QTL region which was reported to be responsible for the number of stillborn piglets in LW and French LR populations by Tribout et al. [64].

The SNP ASGA0072103, located on SSC 16, had chromosome-wide significance in LR_3 and LR_3b. Tribout et al. [64] detected a QTL affecting NBA at this position in LW and French LR populations.

In the same study, a QTL for NBA was found on SSC18 [64]. This supports the findings of the present study. We detected a SNP with genome-wide significance at position 47.3 Mb on SSC18 with a MAF of 0.6%. The results reported by Tribout et al. [64] and our own findings indicate that this chromosome region may have an impact on NBA in Large White populations.

## Conclusion

A distinct genetic stratification between different pig breeds and pig sub-populations was detected in our data set. This might be characteristic for commercial pig populations from competing pig breeding organisation with different breeding goals.

In summary, we found 17 different SNPs in the various sub-clusters. Five of the SNPs had a low MAF (<1%). Taking into account the long selection history for fertility traits and the low heritability of NBA, this result was to be expected. Most of the significant SNPs were detected in chromosome regions where candidate genes or QTL affecting litter size had been mapped in previous studies. Against this background, the removal of SNPs with a low MAF jeopardises the potential for genetic progress in genomic selection programs. Because of the low MAF of many QTL, the probability of finding many SNPs which act as QTL across breeds or sub-clusters was low. This assumption was supported by the low number of across sub-cluster QTL in our study. It appears that in each sub-population litter size is influenced by different alleles. Because there are no such overlapping QTL regions, it is questionable if the combination of genetically divergent sub-populations is a useful strategy for detecting relevant QTL or improving the accuracy of genomic selection.

## Supporting Information

S1 Fig Manhattan plot of genome wide association study for NBA in LW_2. (TIFF)

S2 Fig Manhattan plot of genome wide association study for NBA in LW_2a. (TIFF)

S3 Fig Manhattan plot of genome wide association study for NBA in LW_2b. (TIFF)

S4 Fig Manhattan plot of genome wide association study for NBA in LW_3. (TIFF)

S5 Fig Manhattan plot of genome wide association study for NBA in LR_1. (TIFF)

S6 Fig Manhattan plot of genome wide association study for NBA in LR_2. (TIFF)

S7 Fig Manhattan plot of genome wide association study for NBA in LR_3. (TIFF)

S8 Fig Manhattan plot of genome wide association study for NBA in LR_3a. (TIFF)

S9 Fig Manhattan plot of genome wide association study for NBA in LR_3b. (TIFF)
