# Supplementary figures and images for: A Genome-Wide Association Study in Large White and Landrace Pig Populations for Number Piglets Born Alive
Source: PLoS One. 2015 Mar 17;10(3):e0117468. doi: 10.1371/journal.pone.0117468 (PMC4363374; doi:10.1371/journal.pone.0117468)

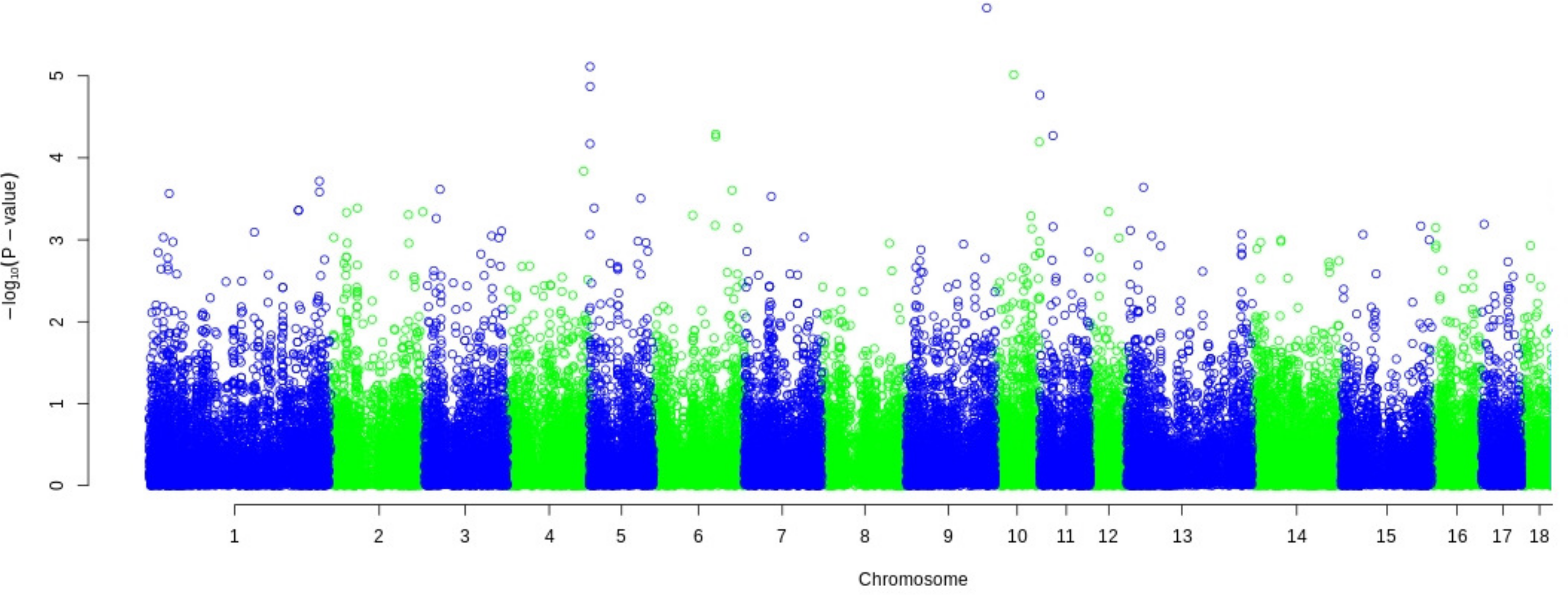

Supplement: S1 Fig — (TIFF) [file pone.0117468.s001.tiff]

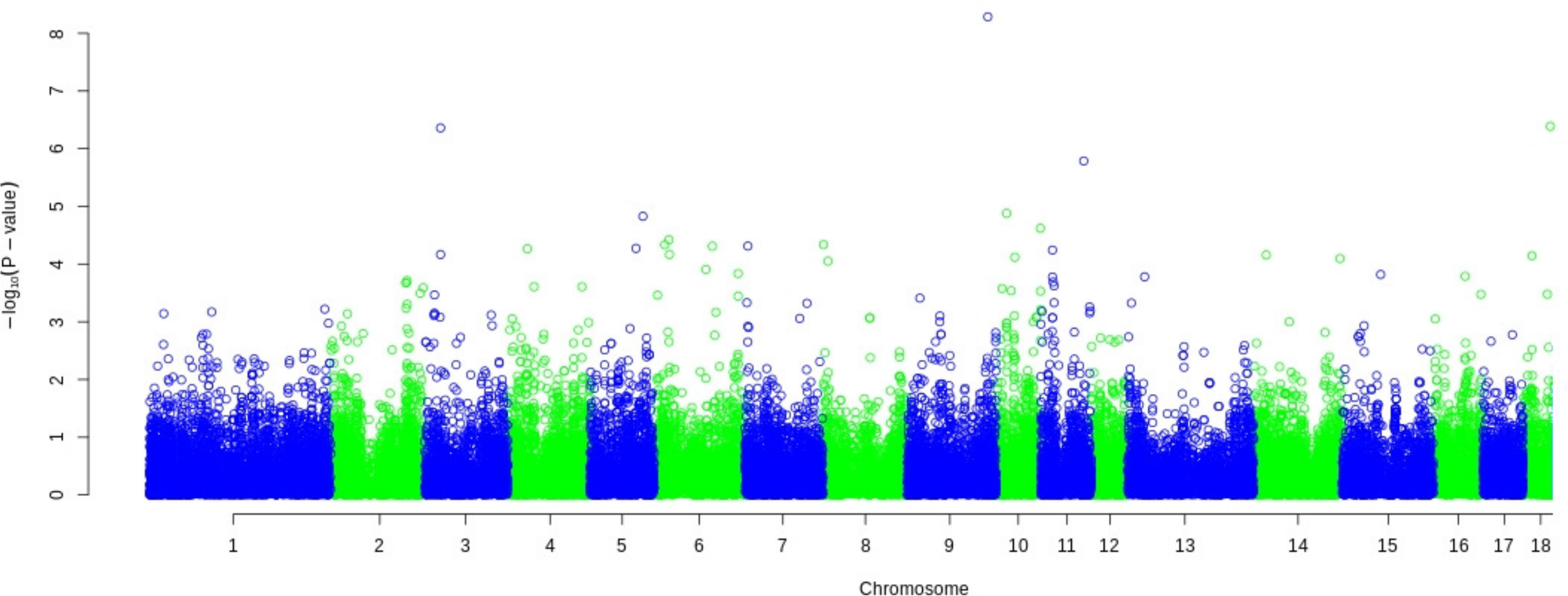

Supplement: S2 Fig — (TIFF) [file pone.0117468.s002.tiff]

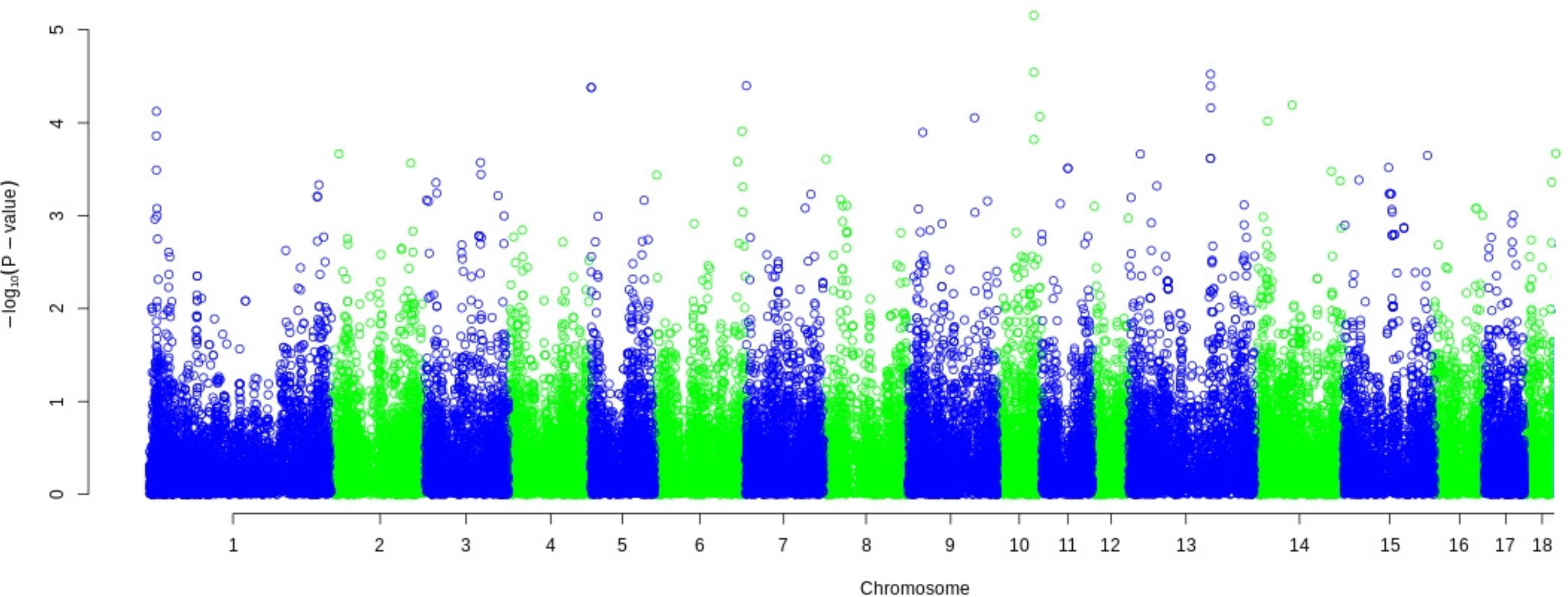

Supplement: S3 Fig — (TIFF) [file pone.0117468.s003.tiff]

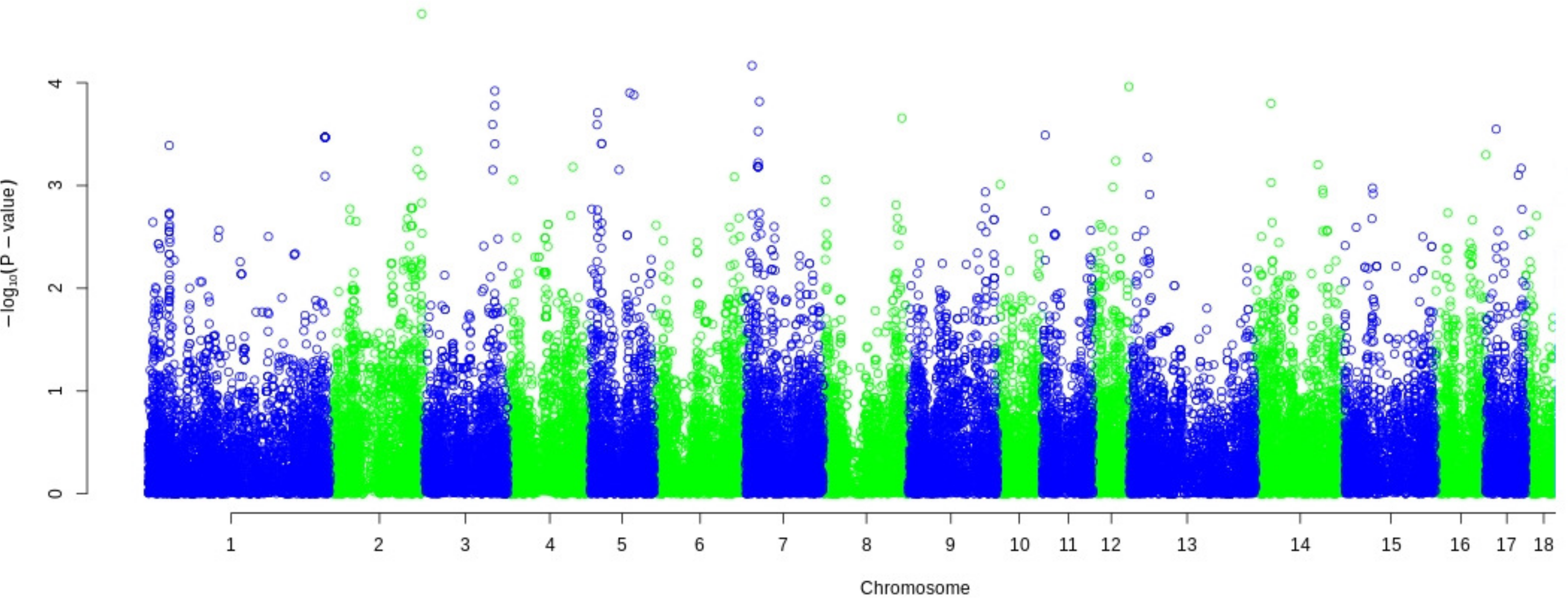

Supplement: S4 Fig — (TIFF) [file pone.0117468.s004.tiff]

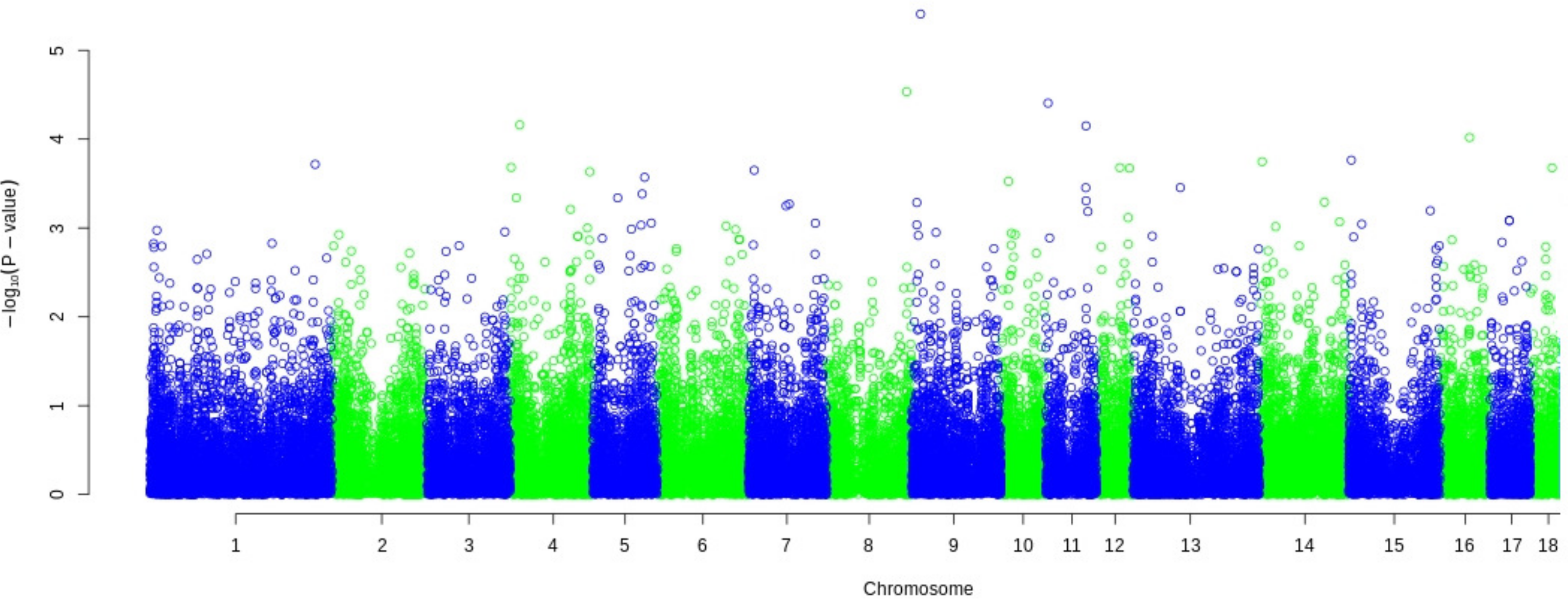

Supplement: S5 Fig — (TIFF) [file pone.0117468.s005.tiff]

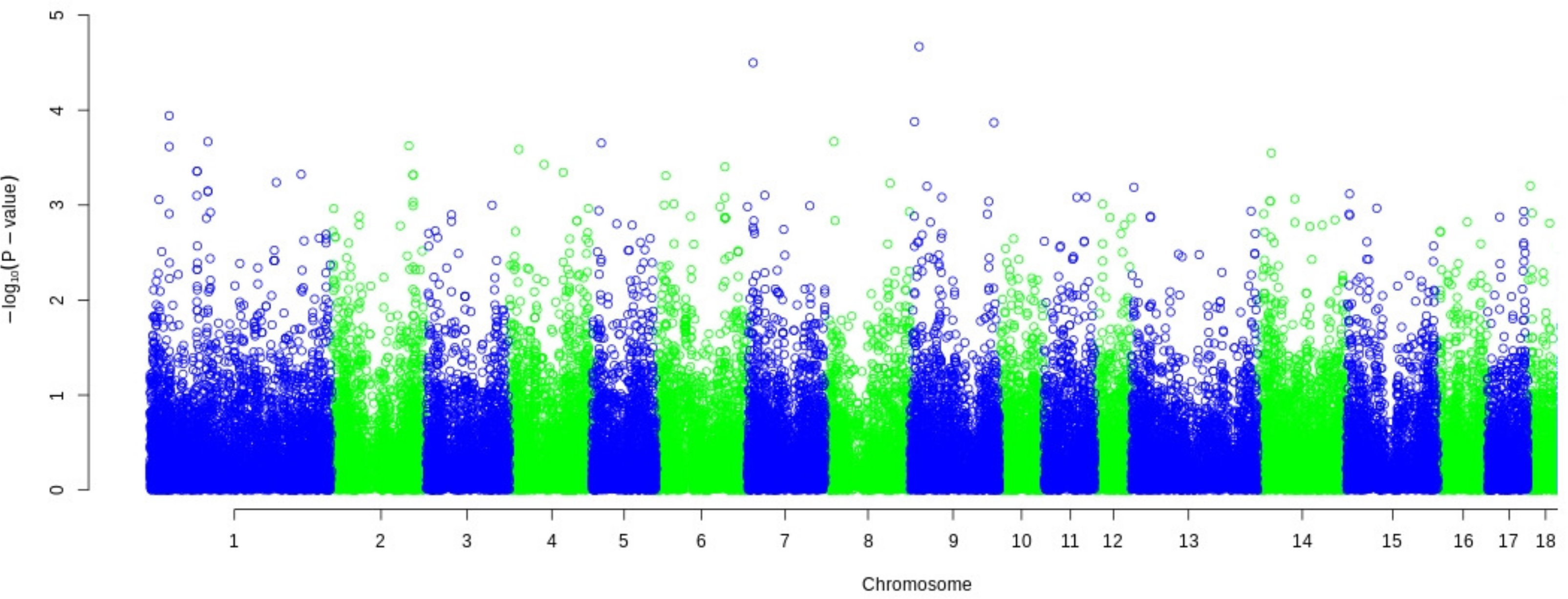

Supplement: S6 Fig — (TIFF) [file pone.0117468.s006.tiff]

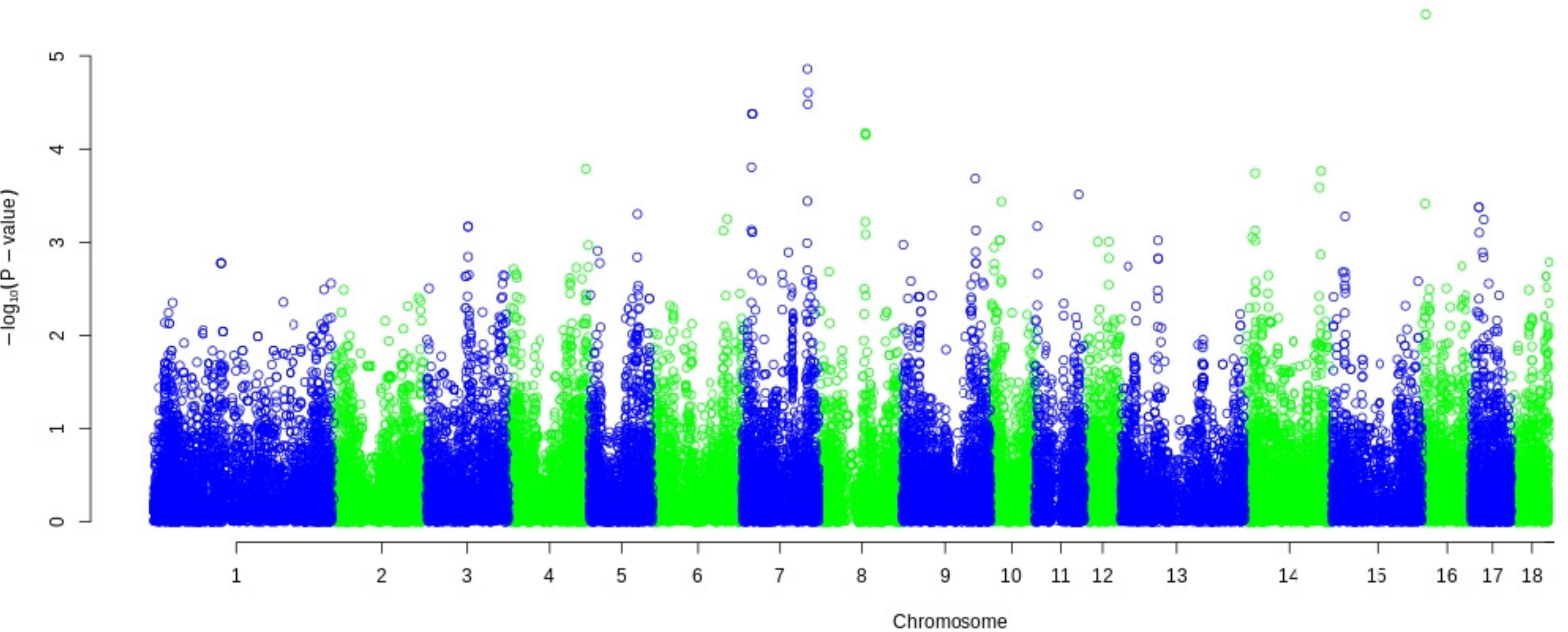

Supplement: S7 Fig — (TIFF) [file pone.0117468.s007.tiff]

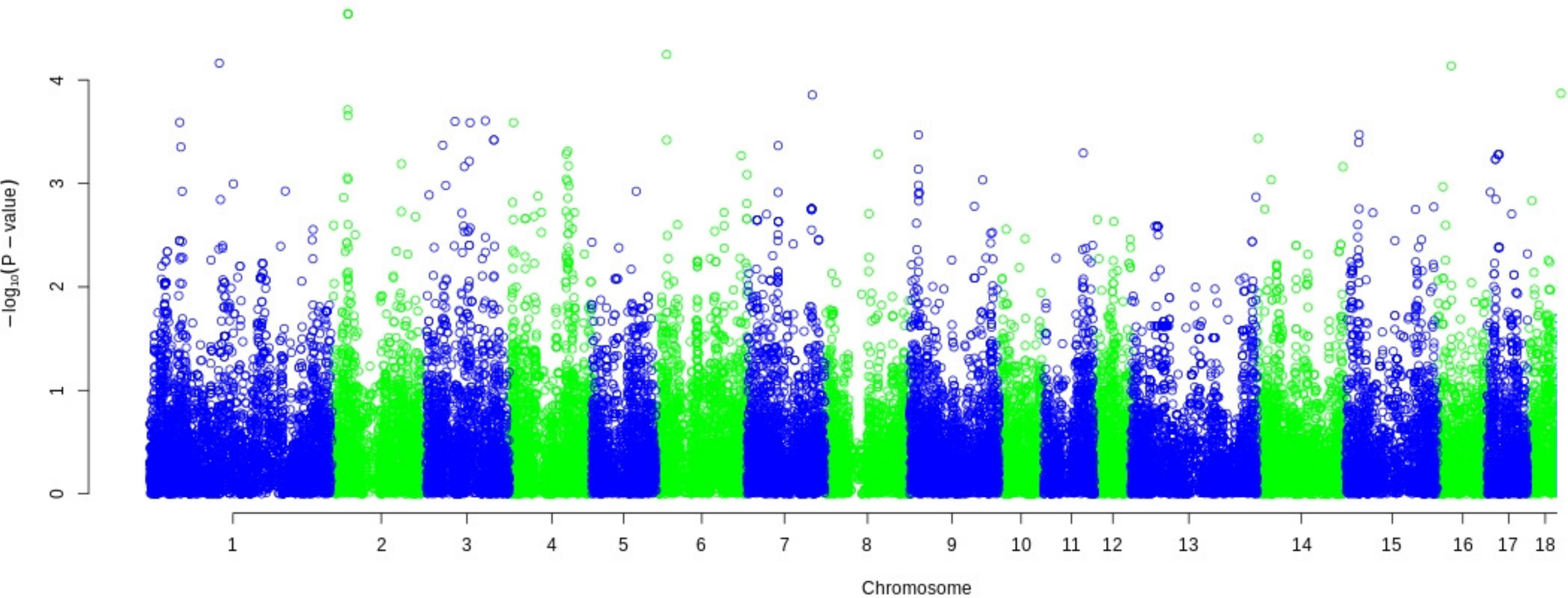

Supplement: S8 Fig — (TIFF) [file pone.0117468.s008.tiff]

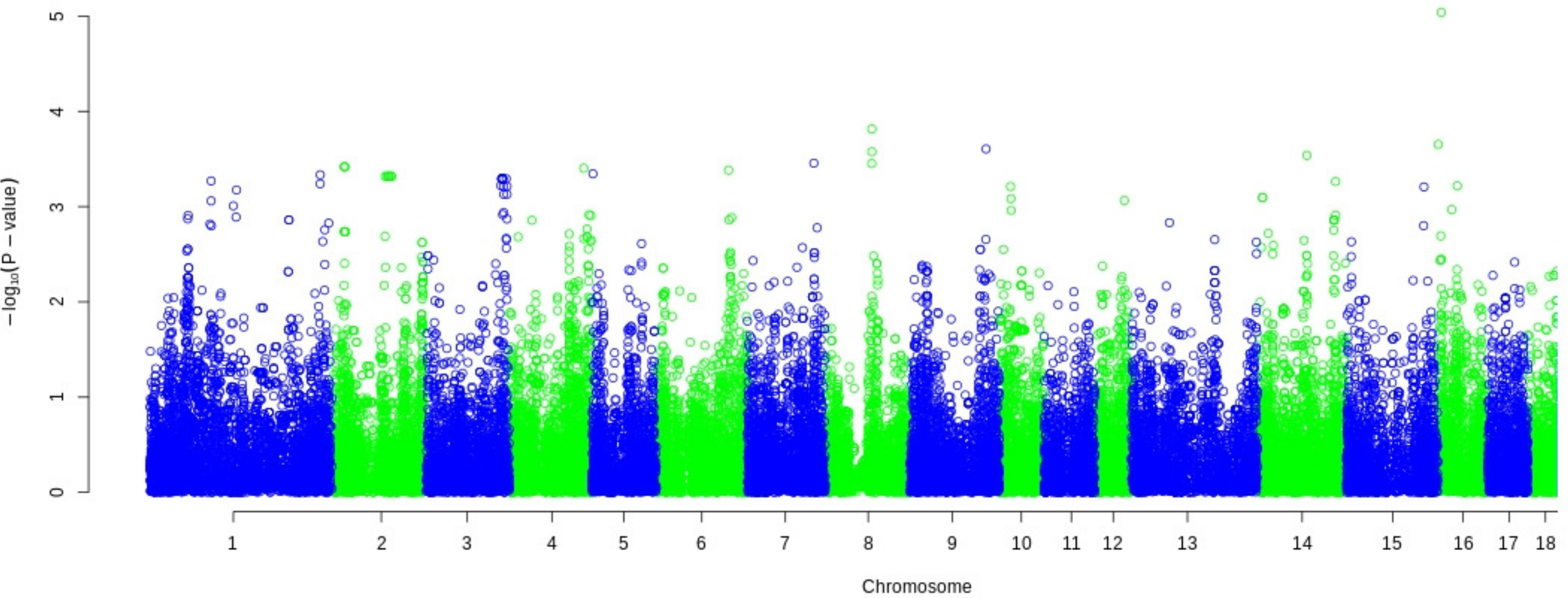

Supplement: S9 Fig — (TIFF) [file pone.0117468.s009.tiff]
